# Production of a *Shigella sonnei* Vaccine Based on Generalized Modules for Membrane Antigens (GMMA), 1790GAHB

**DOI:** 10.1371/journal.pone.0134478

**Published:** 2015-08-06

**Authors:** Christiane Gerke, Anna Maria Colucci, Carlo Giannelli, Silvia Sanzone, Claudia Giorgina Vitali, Luigi Sollai, Omar Rossi, Laura B. Martin, Jochen Auerbach, Vito Di Cioccio, Allan Saul

**Affiliations:** 1 Sclavo Behring Vaccines Institute for Global Health S.r.l., Siena, Italy; 2 Novartis Vaccines and Diagnostics, S.r.l., Siena, Italy; University of Melbourne, AUSTRALIA

## Abstract

Recently, we developed a high yield production process for outer membrane particles from genetically modified bacteria, called Generalized Modules of Membrane Antigens (GMMA), and the corresponding simple two step filtration purification, enabling economic manufacture of these particles for use as vaccines. Using a *Shigella sonnei* strain that was genetically modified to produce penta-acylated lipopolysaccharide (LPS) with reduced endotoxicity and to maintain the virulence plasmid encoding for the immunodominant O antigen component of the LPS, scale up of the process to GMP pilot scale was straightforward and gave high yields of GMMA with required purity and consistent results. GMMA were formulated with Alhydrogel and were highly immunogenic in mice and rabbits. In mice, a single immunization containing 29 ng protein and 1.75 ng of O antigen elicited substantial anti-LPS antibody levels. As GMMA contain LPS and lipoproteins, assessing potential reactogenicity was a key aspect of vaccine development. In an *in vitro* monocyte activation test, GMMA from the production strain showed a 600-fold lower stimulatory activity than GMMA with unmodified LPS. Two *in vivo* tests confirmed the low potential for reactogenicity. We established a modified rabbit pyrogenicity test based on the European Pharmacopoeia pyrogens method but using intramuscular administration of the full human dose (100 μg of protein). The vaccine elicited an average temperature rise of 0.5°C within four hours after administration, which was considered acceptable and showed that the test is able to detect a pyrogenic response. Furthermore, a repeat dose toxicology study in rabbits using intramuscular (100 μg/dose), intranasal (80 μg/dose), and intradermal (10 μg/dose) administration routes showed good tolerability of the vaccine by all routes and supported its suitability for use in humans. The *S*. *sonnei* GMMA vaccine is now in Phase 1 dose-escalation clinical trials.

## Introduction

Shigellosis is a major global health problem, responsible for more than 7 million Disability-Adjusted Life Years and 100,000 deaths per year, especially in children under 5 years old in developing countries [1,2,3]. Shigellosis is caused by Gram-negative bacteria of the genus *Shigella*, which is divided into 4 serogroups and further differentiated into 50 serotypes based on the structure and composition of the outer polysaccharide antigen (O antigen, OAg) of the lipopolysaccharide (LPS): *S*. *sonnei* (1 serotype), *S*. *flexneri* (15 serotypes), *S*. *boydii* (19 serotypes) and *S*. *dysenteriae* (15 serotypes) [4]. A limited number of serotypes contribute to the global burden of disease and these vary between regions and over time [4,5,6,7]. *Shigella sonnei* and *Shigella flexneri* 2a are the currently dominant serotypes worldwide [4,6]. *S*. *sonnei* displaces other serotypes as socioeconomic status improves [7] and is the most frequent cause of traveler’s shigellosis [8]. Antibiotic resistance is increasing globally, limiting treatment options in the effected populations [9,10,11]. Thus, vaccine development remains a high priority.

Natural infection leads to good protective immunity [12]. Protection is highly specific for the infecting serotype [13] suggesting that the dominant protective antigen is the OAg of the LPS. Consistent with this observation, *Shigella* infection leads to a marked rise in anti-LPS serum IgG in convalescent patients [14] and individuals with a high level of anti-LPS serum antibodies are significantly less likely to become infected than subjects with low levels of serum antibody [15]. Multiple approaches have been taken to develop a *Shigella* vaccine: live-attenuated strains, killed whole-cell oral vaccines, and subunit vaccines [5,16,17,18]. Most of these approaches target the serotype-specific OAg. In addition, approaches targeting conserved proteins to achieve serotype-independent protection are in pre-clinical development [19]. The most successful recent vaccine candidate, a parenteral *S*. *sonnei* OAg conjugate, showed 74% protection against homologous *S*. *sonnei* infection in young adults after one immunization [20] and 71% efficacy in children older than 3 years after two immunizations [21]. In contrast, the vaccine showed low immunogenicity and lack of protection in children younger than 3 years [21]. The level of protection paralleled the level of the OAg vaccine-specific antibody response, measured as antibody response to *S*. *sonnei* LPS with the homologous OAg (anti-LPS response) [21]. Thus, a vaccine that could generate stronger responses to the OAg, especially in young children, may make an important public health contribution.

Due to the serotype-specificity of the antibody response, a broadly protective *Shigella* OAg vaccine will require the combination of several serotypes [4,6]. As the major burden of disease is in developing countries, this requires the production of an affordable vaccine.

Recently, we described the development of a vaccine platform based on Generalized Modules for Membrane Antigens (GMMA) [22,23]. GMMA are outer membrane particles shed from Gram-negative bacteria that are genetically modified to enhance the level of particle production. In the case of *Shigella*, the required genetic modification is deletion of the *tolR* gene [22] whose corresponding protein is involved in linking the inner and outer membranes. Similar to native outer membrane vesicles that are naturally shed by Gram-negative bacteria [24], GMMA consist of outer membrane lipids, outer membrane proteins, and soluble periplasmic components. They are highly immunogenic in small doses in animals [22].

As GMMA are derived from the outer membrane of Gram-negative bacteria, they contain several stimulators of the innate immune system [25], especially lipopolysaccharide (LPS) [26] and lipidated proteins. Thus, addressing potential causes of reactogenicity is an important aspect during vaccine development. Previously, we examined ways of reducing the endotoxicity of GMMA by genetically modifying the LPS. The most endotoxic form of LPS is hexa-acylated. Deletion of the late acyltransferase genes *htrB* [27] or *msbB* [28] in *S*. *sonnei* resulted in GMMA with penta-acylated LPS [25]. Using the *in vitro* monocyte activation test (MAT) which has been used to estimate human responses and predict the safety of vaccines, e.g. native outer membrane vesicle vaccine candidates for *Neisseria meningitidis* [29], we demonstrated that GMMA with penta-acylated LPS have markedly reduced potential to induce inflammatory cytokines from human peripheral blood monocytes (PBMC) compared to GMMA with hexa-acylated LPS [25]. The monocyte activation test (MAT) has been developed as alternative to the rabbit pyrogenicity test and different tests have been validated [30] but each required a product-specific validation to replace European Pharmacopeia (Ph.Eur.) rabbit intravenous pyrogenicity test [31]. This test is designed to ensure absence of endotoxins originating from bacterial contaminants. For products derived from Gram-negative bacteria, where a certain level of LPS can be an integral part of the product, the test can only be applied after dilution of the test material to much less than the human dose [32]. Therefore, there is need for an assay that more closely mimics human immunization with products that contain LPS.

In this paper, we describe the GMP production of a GMMA based *S*. *sonnei* vaccine, called 1790GAHB. Although this contains all of the *S*. *sonnei* outer membrane proteins and, thus, there are potentially several specificities through this may induce protective immunity, development of this vaccine is based on the hypothesis that an antibody response to the specific OAg as seen with the conjugate vaccines may be sufficient to give protection. We show that 1790GAHB is highly immunogenic in mice and rabbits generating strong responses to *S*. *sonnei* LPS with homologous OAg and is acceptable for use in human trials as judged by release testing including a modified *in vivo* rabbit pyrogenicity test and GLP rabbit toxicology study.

## Materials and Methods

### Bacterial strain generation, growth conditions, cell banking, and flow cytometry

*S*. *sonnei* 53G [33] was chosen as parent strain. *S*. *sonnei* strain NVGH1859 (*S*. *sonnei* 53G Δ*tolR*::*kan* Δ*virG*::*nadAB*) was obtained by replacing the plasmid-encoded *virG* gene [34] in *S*. *sonnei* 53G Δ*tolR*::*kan* [22] with the genes *nadA* and *nadB* from *E*. *coli* [35]. The upstream and downstream regions of *virG* were amplified using the primer pairs virGup-5/virGup-3 (upstream) and virGdown-5/virGdown-3 (downstream) (Table 1). The “*nadAB*” cassette was generated by amplifying *nadA* and *nadB* from *E*. *coli* using primers nadA-5/nadA-3 and nadB-5/nadB-3 (Table 1). The fragments were inserted into pBluescript (Stratagene) so that *nadA* and *nadB* were linked and interposed the flanking regions of *virG*. The replacement construct (*virG*up-*nadAB*-*virG*down) was amplified using the primers virGup-5/virGdown-3 and used to transform recombination prone *S*. *sonnei* Δ*tolR*::*kan* as previously described [22].

**Table 1:** Primers used in this study.

Primer	Sequence 5’ → 3’
virGup-5	ACTCGAGCTCTGTAGTTGATTTGACAGTTGACATCC
virGup-3	CTAACCCGGGCACTATATTATCAGTAAGTGGTTGATAAACC
virGdown-5	CTAACCCGGGCGTGTTGATGTCCTGC
virGdown-3	ACGCGTCGACAGTTCAGTTCAGGCTGTACGC
nadA-5*	CTAACCCGGGCAAGCAACTCTATGTCGGTGGAAT
nadA-3*	TATCAAGCTTGGCAAGGCCAATACACAGC
nadB-5*	TATCAAGCTTAGGGTTAGAGTGTCTCGTTTTTGTA
nadB-3*	CTAACCCGGGCCAGACCAGAACTATTCC

*nadA and *nad B* primers as described by Prunier *et al*. [35] with small modifications

*S*. *sonnei* strain NVGH1790 (*S*. *sonnei* 53G Δ*tolR*::*kan* Δ*virG*::*nadAB* Δ*htrB*::*cat*) was generated from NVGH1859 by replacing the *htrB* gene [27] by the chloramphenicol resistance gene *cat* as described by Rossi *et al*. [25].

NVGH1790 was routinely cultured in *S*. *sonnei* Defined Medium (SSDM, [22]) with glucose as carbon source. The SSDM was prepared as follows: 13.3 g/kg KH_2_PO_4_, 4 g/kg (NH_4_)_2_HPO_4_, 1.7 g/kg citric acid monohydrate, 2.5 g/kg L-aspartic acid, 493 mg/kg MgSO_4_*H_2_O, 2.7 mg/kg Co(NH_3_)_6_Cl_3_, 15 mg/kg MnCl_2_*4H_2_O, 1.5 mg/kg CuCl_2_*H_2_O, 3 mg/kg H_3_BO_3_, 2.5 mg/kg Na_2_MoO_4_*2H_2_O, 2.5 mg/kg zinc acetate monohydrate, 0.49 mg/kg ferric citrate, 50 mg/kg thiamine. Glucose was added at a concentration of 5 g/kg for solid medium and shake flask cultures. For growth in fermenters, 27.3 g/kg glucose and 0.25 g/kg polypropylene glycol (PPG) were added. Solid SSDM contained 18 g/L agar.

### Cell banking

To minimize the risk of contamination with TSE or adventitious agents, the cell line was cleaned under GMP by three passages on agar plates prepared with SSDM. GMP master and working cell banks were prepared.

### Flow cytometry

Cells from fermenter or flask scale culture were preserved for flow cytometry analysis by fixation in 0.5% formaldehyde. 9x10^5^ cells were stained with *S*. *sonnei* Phase I monovalent rabbit antiserum (Denka Seiken Co., Ltd.), reacting with the O antigen (OAg) of *S*. *sonnei*, and bound antibodies were detected using Fluorescein-conjugated F(ab’)_2_ fragment goat anti-rabbit IgG (Jackson ImmunoResearch Europe Ltd.). Samples were fixed using 4% formaldehyde and analyzed using a FACScanto II flow cytometer (BD Biosciences). The data were processed using FlowJo software (Tree Star Inc.).

### GMMA production

#### Fermentation

For each production batch, the NVGH1790 inoculum was grown in a shake flask from the research or GMP cell bank in SSDM at 30°C with agitation (200 rpm), starting from an optical density measured at 600 nm (OD_**600**_) of 0.02 until the culture reached an OD_**600**_ equal to 1.5 ± 0.5, usually in 9 ± 2 hours. In the bioreactor (30 L scale in Sartorius, Biostat D75 Bioreactor, or 25 L scale in LP35 Bioengineering Bioreactor), NVGH1790 was cultured in batch mode starting from 2% inoculum size with controlled cultivation conditions: pH 6.7 kept by addition of 28% NH_**4**_OH, 30°C, dissolved oxygen kept at 30% saturation by 1 air volume per culture volume per minute (vvm) airflow and agitation in cascade (200–800 rpm) until the final OD_**600**_ of 35.

#### Purification

GMMA produced from strain NVGH1790 are called 1790-GMMA. GMMA released into the fermentation broth were purified using two consecutive Tangential Flow Filtration (TFF) steps: a microfiltration in which the culture supernatant containing the GMMA is separated from the bacteria, and an ultrafiltration, in which the GMMA were separated from soluble proteins. For the microfiltration step (1.2 m^**2**^ of a 0.2 μm pore size cellulose membrane) the bioreactor was connected with the TFF system, in order to use the fermentation vessel as a recirculation tank. The culture supernatant was initially concentrated three times to reach “one volume” of concentrated supernatant, followed by a discontinuous diafiltration against five volumes of the buffer in the growth medium (13.3 g/kg of KH_**2**_PO_**4**_; 4 g/kg of NH_**4**_HPO_**4**_; 1.7 g/kg of Citric acid; 4 mL/L of NH_**4**_OH; pH 6.7). The microfiltered material, containing GMMA, was then filtered through a filter capsule with 0.45 μm then 0.2 μm filters (Sartorius) to ensure absence of any viable *Shigella* bacteria before further processing. The ultrafiltration step (1.4 m^**2**^ of a 300 kDa pore size PES membrane) consisted of a continuous diafiltration of the microfiltered GMMA solution against ten volumes of Tris-buffered saline (TBS), 0.9% NaCl,10 mM Tris/Tris HCl pH 7.4, and permitted substantial removal of nucleic acids and soluble proteins. A final concentration of the purified GMMA was performed to obtain the required concentration for the formulation process and filtered through a Sartorius cellulose acetate sterilizing filter which was validated for extractables, leachables, and bacteria retention capability with GMMA bulk. Three non-GMP consistency batches were prepared from a fermentation volume of 30 L. One of these batches NVGH1883 was used as reference GMMA and stored in small volumes at -80°C. The studies in this paper also used a single GMP batch (batch number 1112) prepared from a fermentation volume of 25 L.

### Formulation

#### GMP formulation of 1790GAHB

1790-GMMA were adsorbed to aluminum hydroxide (Alhydrogel 2%, Brenntag Biosector, Denmark) by adding the GMMA suspension to Alhydrogel under constant stirring at room temperature for 2 h followed by storage at 2–8°C overnight. The 1790-GMMA Alhydrogel formulation, called 1790GAHB, contains 200 μg/mL GMMA protein and 0.7 mg/mL aluminum-III-ion (Al^**3+**^) as Alhydrogel in TBS. 1790GAHB was dispensed at 0.7 mL per 3 mL single dose vial. The formulation was tested for identity, total protein content, aluminum content, extractable volume, non-adsorbed protein, visual appearance, pH, osmolality, sterility, immunogenicity, and pyrogenicity. Two GMP lots: a toxicology lot and a clinical lot were prepared. A smaller (140 mL) non-GMP stability lot was also generated. Freshly formulated small scale laboratory batches were produced for initial pyrogenicity and immunogenicity studies.

#### GMP formulation of GAHB-Placebo

Placebo was prepared containing 0.7 mg/mL Al^**3+**^ as Alhydrogel in TBS and was dispensed at 0.7 mL per 3 mL vial. The GAHB-Placebo was tested for identity, aluminum content, extractable volume, visual appearance, pH, osmolality, sterility and pyrogenicity.

### Physico-Chemical Analytical Methods

#### Protein quantification of 1790-GMMA and 1790GAHB

Protein quantification was routinely performed by Lowry assay. For determining protein concentration in unadsorbed GMMA, assays used a secondary BSA standard calibrated as previously described [36] against a primary 1790-GMMA standard with a protein content determined by quantitative amino acid analysis. Thus all GMMA protein concentrations are indirectly referenced to the protein concentration determined by amino acid analysis. Samples containing Tris were diluted to a final Tris concentration of equal to or less than 1 mM to avoid interference in the Lowry assay.

For protein quantification of GMMA adsorbed to Alhydrogel, e.g. 1790GAHB, the secondary BSA standard was adsorbed to Alhydrogel. After the color development, the samples were centrifuged and the absorbances of the supernants were determined.

Soluble protein in unadsorbed GMMA preparations was determined in the supernatant following ultracentrifugation at 186,000 g at 4°C for 2 h using a secondary BSA standard calibrated against a primary soluble protein standard quantified by quantitative amino acid analysis.

The unbound protein content in GMMA adsorbed to Alhydrogel was too low to measure by Lowry and was assessed by SDS-PAGE (10% bis-acrylamide gel) of supernatants collected after centrifugation of the sample and compared with reference 1790-GMMA run in a parallel lane. Protein bands were visualized by silver staining, quantified by densitometry, and the data analyzed using ImageScanner III software. The intensity of detectable bands in the supernatant sample was compared (as limit test) to the intensity of the corresponding bands in the 1790-GMMA sample. The limit corresponds to 5 **μ**g/mL of non-adsorbed protein (2.5% of total protein of the 1790GAHB formulation).

#### GMMA protein profile

1790-GMMA were denatured for 10 min at 90°C in sodium dodecyl sulfate-polyacrylamide gel electrophoresis (SDS-PAGE) sample buffer containing SDS (Invitrogen, LDS NuPAGE sample buffer) and 50 mM dithiothreitol (DTT). 3 μg of protein were loaded onto a 10% (wt/vol) polyacrylamide gel (Invitrogen, Novex 10%). Electrophoresis was carried out in 3-(N-morpholino)-propanesulfonic acid (MOPS) buffer (Invitrogen) at 40 mA for 75 min. The separated proteins were stained with brilliant Blue G-colloidal Coomassie (Sigma-Aldrich) and analyzed using densitometry.

#### O antigen quantification

For detailed characterization and lot release assays, the O antigen (OAg) concentration was measured by a combination of NMR and HPAEC-PAD analysis. The sugar content of both the core and the repeating units was determined as follows:

*Core quantification (OAg and LPS)*: Quantitative determination of the core sugar galactose by HPAEC-PAD analysis [37] was used to quantify the amount of LPS in 1790-GMMA based on two galactose residues per core [38].

*O antigen reference standard*: GMMA from *S*. *sonnei* NVGH1859 (1859-GMMA) were hydrolyzed for 2 h at 100°C in 1% acetic acid. The cleaved lipid A and precipitated protein were both removed by centrifugation [37]. The main fraction (medium molecular weight, MMW) of OAg was recovered by Size Exclusion Chromatography (SEC, GE Sephacryl S-100HR column). The OAg size was determined by measuring the ratio of repeating units by ^1^H-NMR [38]. The average was 33. The O antigen quantity was determined from the average number of repeating units and the molar concentration of the LPS core by galactose measured by HPAEC-PAD analysis as described above.

*O antigen quantification in GMMA*: LPS was purified by hot phenol-water extraction [39] as follows: 400 **μ**L of sample was added to an equal volume of 90% phenol pH 8 solution (Sigma-Aldrich) and incubated at 80°C for 2 h. After cooling to 4°C, the sample was centrifuged at 18000 g at 4°C for 15 min and the upper water phase containing the LPS was recovered. The phenol phase was extracted again with 400 **μ**L of water (1 h at 80°C) and after centrifugation (18000 g at 4°C for 15 min) the water phase was recovered, combined with the first water extract, and dried overnight in a centrifugal evaporator. The sample was reconstituted in water (400 **μ**L) and eventually diluted with water to give a concentration of O antigen between 2.5 and 30 **μ**g/mL. LPS yield was >90% as judged by the recovery of LPS core, measured using galactose as described above. The LPS was subjected to alkaline hydrolysis and sugars were measured by HPAEC-PAD analysis as reported for Vi polysaccharide [40] and compared with the O antigen reference standard.

#### LPS size distribution

For analysis by SDS-PAGE, LPS was purified using the phenol-water method (see above) with modifications. GMMA (1 mg/mL) were boiled for 3 min, incubated with 0.5 μg/μL proteinase K (Sigma Aldrich) at 60°C overnight, mixed 1:1 (vol/vol) with saturated phenol pH 8.0 (Sigma Aldrich), incubated for 30 min at 70°C, and centrifuged for 1 h at 10,000 g at room temperature. The upper phase was recovered, mixed 2:1 (vol/vol) with 100% ethanol, and LPS was precipitated for 1 h at -80°C and pelleted by centrifuging at 12,000 g for 30 min at room temperature. The pellet was dried using a SpeedVac and dissolved in water. LPS was electrophoresed on 12% bis-tris polyacrylamide gel (Life technologies) and stained using the Silver Quest Silver Staining Kit (Life Technologies).

#### MALDI-TOF analysis of lipid A

Lipid A was precipitated from GMMA as previously described [25] using mild acid hydrolysis with 1% acetic acid for 2 h at 100°C. Samples were centrifuged at 14,000 g for 15 min, the pellets resuspended in water, and washed twice with water. The pellets were dried overnight using a Speedvac, resuspended in chloroform-methanol 4:1, and mixed with an equal volume of Super DHB (Sigma-Aldrich) solution in water/acetonitrile 1:1 (vol/vol). Two μL of the mixture were loaded to the target plate (MTP 384 target plate ground steel BC, Bruker Daltonics) and analyzed by Ultraflex MALDI-TOF (Bruker Daltonics) in reflectron ion-negative mode. A Peptide Calibration Standard (Bruker Daltonics), mixed with the Super DHB solution, was included in each analysis. The *m/z* rations were determined by Flex Analysis software in comparison to the Peptide Standard.

#### GMMA particle size

*Dynamic light scattering*: The size distribution of 1790-GMMA was evaluated by dynamic light scattering using a Malvern Zetasizer Nano ZS. The particle size distribution was obtained as intensity of the scattered light using the Z-average value of three different measurements of the 173° backscattered light with “protein” as material setting and “General purpose (normal resolution)”.

*Negative Staining Transmission Electron Microscopy of GMMA*: GMMA were prepared and observed by electron microscopy as previously described [25]. Electron micrographs were recorded at a nominal magnification of 105,000 X. GMMA diameters were measured manually on printed copies of the electron micrographs in comparison to the scale bar.

### Biological assays

#### PBMC isolation and MAT

The *in vitro* Interleukin 6 (IL-6) production of PBMC following stimulation with GMMA was used as *in vitro* surrogate to assess reactogenic potential using the procedure described by Rossi et al. [25]. Briefly, buffy coats from different donors were used to isolate PMBC using Ficoll density centrifugation as reported [29]. PBMC were seeded at a density of 2x10^**5**^ cells/well with 180 μL of RPMI-1640 complemented with 25 mM HEPES, 2 mM glutamine, 10% FBS, 1% Pen-Strep (InvitroGen) in 96-well round bottom plates. 20 μL of 10-fold serial dilutions of GMMA in TBS (0.0001–1,000 ng/mL final concentration in the assay) were added, cells were incubated for 4 h at 37°C, and supernatants were recovered after centrifugation (5 min, 400 g) and stored at -80°C until analyzed for IL-6 concentration.

#### Immunogenicity/potency studies in mice

Eight BALB/c mice per group (female, 4 to 6 weeks old) received one or two intraperitoneal injections of different doses of 1790GAHB on days 0, and 21 in a volume of 0.5 mL. Control mice received 0.5 mL of GAHB-Placebo. Blood samples were collected on days 7, 14, 21, 28, and 35, or in some studies only on day 21. In potency assays, groups of mice were immunized with four different doses of the vaccine or reference standard: 1790-GMMA reference batch stored at -80°C then formulated freshly on Alhydrogel.

#### Enzyme-linked immunosorbent assay (ELISA)

Antibodies elicited to *S*. *sonnei* LPS are assessed by ELISA using *Shigella sonnei* LPS as plate coating antigen. Nunc Maxisorb 96-well plates were coated over night at 2–8°C with 2 μg/mL LPS, purified from *S*. *sonnei* strain NVGH1859, in phosphate-buffered saline (PBS). The plates were blocked for 2 h with 5% milk in PBS and subsequently washed three times with PBS containing 0.05% Tween 20 (PBST). Mouse sera were diluted 1:100 and 1:4000 in PBST with 0.1% BSA, rabbit sera were diluted in 5% milk in PBS. Diluted sera were incubated in triplicate for 2 h in the ELISA pates. The samples were tested in comparison to previously established and calibrated anti-*S*. *sonnei* LPS standard sera included in a duplicate series of dilutions on each of the plates. After incubation with sample and reference sera the plates were washed three times as above. Bound antibody was detected using a goat anti-mouse IgG or goat anti-rabbit IgG conjugated to alkaline phosphatase, diluted in PBS, 0.1% BSA, 0.05% Tween-20, and followed by three washing steps and a color reaction with p-nitrophenyl phosphate substrate. After 1 h, absorbance (optical density, OD) was measured at 405 nm and 490 nm wavelength and the OD405nm-490nm was calculated. Results are expressed in ELISA units determined relative to the standard serum. One ELISA unit equals the reciprocal of the dilution of the standard serum giving an OD405nm-490nm of 1 in the standard assay.

#### Pyrogenicity

We established a modification of the European Pharmacopeia intravenous pyrogenicity test method (Ph.Eur. 2.6.8 pyrogens, [31]) using the administration of a full human dose delivered intramuscularly. Two sets of experiments were carried out to establish the assay. In the first experiment (under non-GMP conditions but in the GMP facility), three groups of 3 rabbits, preselected according Ph.Eur. 2.6.8 pyrogens, were placed in retaining boxes and the body temperatures were recorded using a rectal probe and the initial temperature was determined. The toxicology lot of the vaccine (0.5 mL) was injected intramuscularly to each of three rabbits in two vaccine groups and 0.5 mL sterile physiological saline to the three rabbits of the control group. Temperature was recorded continuously by an automated system from 90 min before injection until 3 h after administration to determine the initial temperature and a possible temperature rise after administration. Temperature was recorded manually at 3.5, 4, 5, 5.5, 6, 6.5 and 7 h. The next day, the rabbits were placed back in the retaining box, allowed to acclimatize and another reading taken at 24 h.

On the basis of the data (see Results), the following test was chosen for the intra-muscular pyrogenicity test for 1790GAHB. Two groups of three 3 rabbits (one vaccine test and one control group) are selected according Ph.Eur. 2.6.8, placed in retaining boxes, and the initial temperature is determined using a rectal probe. The vaccine (0.5 mL) is administered intramuscularly to rabbits in the vaccine group and 0.5 mL sterile physiological saline to rabbits of the control group. Temperature is recorded continuously by an automated system for 3 h and additional readings are taken manually at 3.5 and 4 h. The maximum temperature rise for each rabbit is determined (the difference between the highest temperature measured during the 4 h period after administration and the initial temperature). For the test to be valid, the mean of the maximum temperature rise of three controls has to be ≤ 0.3°C. The test passes if the mean maximum temperature rise of three vaccine test rabbits is <0.8°C, and fails if the mean maximum temperature rise is ≥ 1.2°C. The test will be repeated if the mean maximum temperature rise of the three rabbits is >0.8 but <1.2°C. For the repeat test in 3 additional rabbits, the test would pass if the mean maximum temperature rise of the three rabbits is ≤0.8°C and otherwise fail.

The second study was carried out under GMP conditions in the GMP facility using the criteria above to assess pyrogenicity of the toxicology and the clinical vaccine lots. The temperature recording in the study was extended over a 24 h period to provide further data on the robustness of the assay and the choice of 4 h as the definitive time period to assess temperature rise.

### Repeat Dose Toxicology study

To support the clinical administration of up to three immunizations of *S*. *sonnei* 1790GAHB vaccine, a toxicology study was conducted with New Zealand White rabbits in compliance with Good Laboratory Practice (GLP) standards (WIL Research Europe, Lyon, France). The vaccine was administered four times, two-weeks apart by the intramuscular (IM), intranasal (IN), or intradermal (ID) clinical route, followed by a two-week observation period. Rabbits were selected as the animal model based on preliminary research studies demonstrating capability to produce an immune response. The study design is presented in Table 2. All animals were observed during the course of the study for morbidity/mortality, clinical observations/examination, Draize injection sites, ophthalmology, body weights, food consumption. Clinical pathology, including coagulation parameters and C-reactive protein (pretest, on day 2, and at both necropsies), antibody analysis (pretest, predose, and at both necropsies), macroscopic observations at necropsies, organs weights, and histopathology (complete WHO tissue list) were also performed in all groups. Body temperatures of groups 2 and 5 (IM) at first immunization were measured at 1.5 h, 0.5 h, and 2 min (0 h) before dosing and at 0.5, 2, 6, and 24 h after injection. The average of the temperature at -0.5 h and 0 h was considered as the initial temperature of the rabbits. At the 2^nd^, 3^rd^, and 4^th^ immunizations of groups 2 and 5 and at all immunizations of groups 1, 3, 4, 6, and 7, body temperatures were recorded prior (0 h), and 2, 6, and 24 h after dosing.

**Table 2:** Experimental design of toxicology study.

					Number of animals necropsied on day 44		Number of animals necropsied on day 56	
Group/ Treatment		Route(s)^a^	Antigen Dose Level (μg protein/ day)	Dose volume (μL/day)	Males	Females	Males	Females
1^b^	0.9% NaCl	IM, IN^c^, ID	0	500, 400,50	4	4	4	4
2	GAHB-Placebo	IM	0	500	4	4	4	4
3	GAHB-Placebo	IN	0	400	4	4	4	4
4	GAHB-Placebo	ID	0	50	4	4	4	4
5	1790GAHB	IM	100	500	4	4	4	4
6	1790GAHB	IN	80	400	4	4	4	4
7	1790GAHB	ID	10	50	4	4	4	4

Dosing days: 0, 14, 28 and 42.

^a^IM: intramuscular; IN: intranasal; ID: intradermal.

^b^Each animal in group 1 (control) received the sterile saline (0.9% NaCl) via all three routes.

^c^4 administrations of 100 **μ**L per nostril 2 h apart, i.e. 400 **μ**L/day. Nostrils were alternated between dosing days.

### Irwin study in rats with IN vaccination

To further support IN administration of 1790GAHB, a GLP Irwin test was undertaken to identify potential undesired effects of 1790GAHB on the central and the peripheral nervous system as judged by a neurobehavioural observation battery [41]. Three groups of 6 male, approximately 0.3 kg, Han Wistar rats were used. The first group received saline control, the second GAHB-Placebo and the third 1790GAHB. Each rat received a single dose of 15 **μ**L in each nostril (total of 30 **μ**L). This volume administered was the maximum practical dose. The test group received 6 **μ**g total of 1790GAHB. On a body weight basis, the 6 **μ**g dose in a 0.3 kg rat is approximately 15 times the highest anticipated dose that would be administered IN to a 60 kg subject in the Phase 1 trial. Rats were monitored, prior to and at and 0.5, 1, 2, 5, and 24 hours post administration.

### Ethics Statement

All animal studies complied with the EU Directive 2010/63 on the protection of animals for scientific purposes, and its implementation in the relevant local laws in Italy and France, respectively. The animal experiments performed at the animal facility of Novartis Vaccines (Siena, Italy) complied with the institutional policies of Novartis and were approved by the Animal Welfare Body (AWB) of Novartis Vaccines, Siena, Italy, and the Italian Ministry of Health (approval numbers AEC 201016, 201018 and 201308). The GLP toxicology and Irwin studies performed at WIL Research Europe (Lyon, France) were reviewed by the ethical committee of WIL Research Europe and the AWB of Novartis Vaccines (Study numbers AB11034 and AB17329). The modified GMP pyrogenicity test was approved by the Charles River France Ethics Committee (Study numbers T 13.1446–48, T 13.1678–80, T 13.1702).

## Results

### O antigen expression of *Shigella sonnei* NVGH1790 cell line

Strain NVGH1790 was genetically modified by the integration of *E*. *coli nadA* and *nadB* genes into the virulence plasmid to remove the nicotinic acid auxotrophy of *Shigella* [35]. Thus, retention of the virulence plasmid and consequently the production of the OAg encoded on the plasmid [42] is ensured by grow in medium without nicotinic acid. Flow cytometry analysis of *S*. *sonnei* NVGH1790 after 25 generations in flasks and after a 30 L fermentation showed that >95% of bacteria were positive for OAg thus showing retention of this plasmid.

### Production at pilot scale and characterization of 1790-GMMA

Three 30 L consistency runs were performed. For each batch, the fermentation process optimized at 30 L was stopped when the OD_600_ was approximately 35, within 20±4 hours from the inoculation of the bioreactor, when a dissolved pO_2_ spike occurred and the pH started increasing. At the end of the fermentation, the culture was harvested by microfiltration. Subsequently, GMMA were purified by ultrafiltration. The process was transferred to an external Contract Manufacturing Organization for production of a GMP lot of 1790-GMMA. The GMP batch was produced at 25 L scale. Data are presented for one of the consistency lot produced at NVGH, 1790-GMMA batch NVGH1883 (reference batch), and for the GMP drug substance, 1790-GMMA batch 1112 (GMP batch).

### Yield and characterization of GMMA

#### Size and integrity

**Fig 1:**
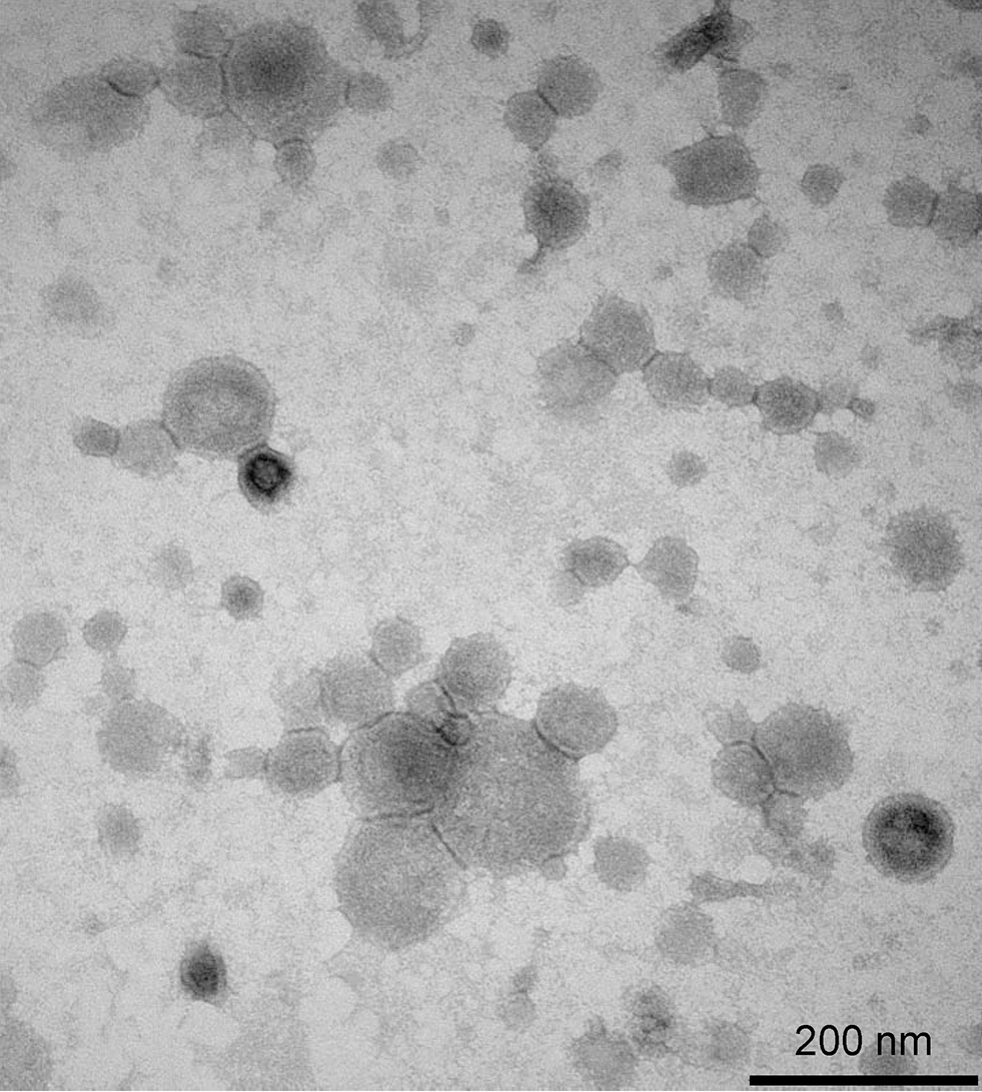
Transmission electron micrograph of purified 1790-GMMA from the GMP batch. The length of the bar in the micrographs is 200 nm.

By electron microscopy, the purified GMMA from reference and GMP batches showed particles with a bimodal size distribution. The majority of the particles are small with an average size of approximately 25–40 nm in diameter. A minor fraction of the particles was larger with sizes between 65 and 140 nm (16% of particles). A representative electron micrograph of the GMP batch is shown in Fig 1.

Dimensional analysis by Dynamic Light Scattering (DLS) using a Malvern Zetasizer Nano gave Z-average 117.0 nm, polydispersion index 0.19; and 114.0 nm, polydispersion index of 0.18 for reference and GMP batches, respectively. Importantly, DLS results were unaltered by multiple freeze thaw cycles or storage at -80°C indicating that the GMMA remained intact and did not aggregate under these conditions. Thus, 1790-GMMA were routinely stored at -80°C prior to formulation.

#### Yield

Reference and GMP batches gave a final yield of 2.4 g protein (from 30 L) and 1.7 g (from 25 L) protein in the purified GMMA, respectively. These lots contained 145 mg and 108 mg of OAg, respectively, with almost identical ratios of OAg to protein (60.3 μg/mg and 63.6 μg/mg). The other two consistency lots (from 30 L) were similar: yield, 2.0 g and 3.2 g; OAg to protein ratio, 61.6 μg/mg and 50.3 μg/mg, respectively.

#### Protein profile

**Fig 2:**
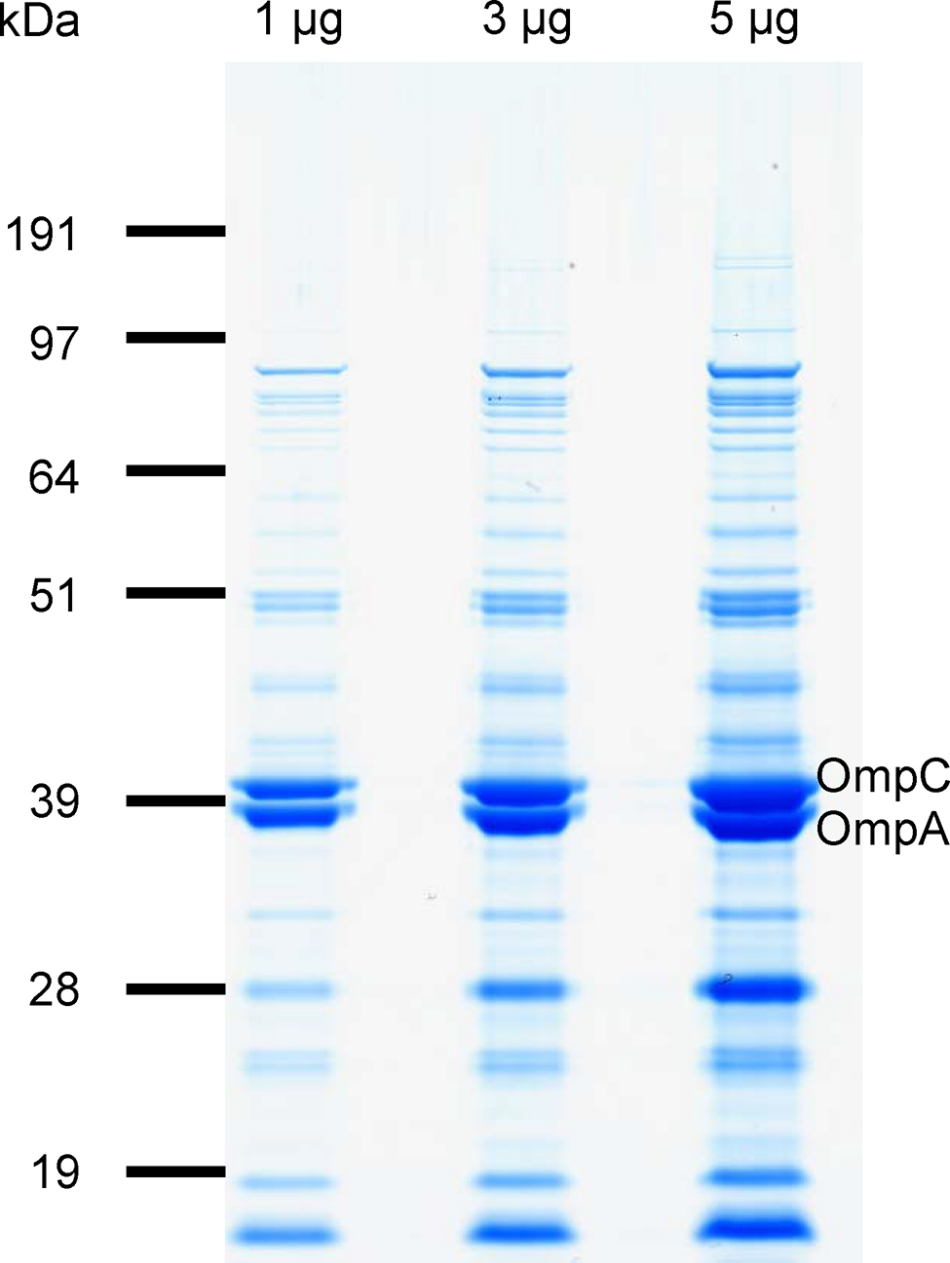
Coomassie Blue stained SDS-PAGE of 1, 3 and 5 μg protein of 1790-GMMA from the reference batch.

The SDS-PAGE profile of 1790-GMMA proteins (Fig 2) was similar to that seen in previous studies [22,36]. The dominant bands at approximately 39 kDa size were identified as OmpA and OmpC by mass spectrometry analysis.

#### LPS and O antigen profile

**Fig 3:**
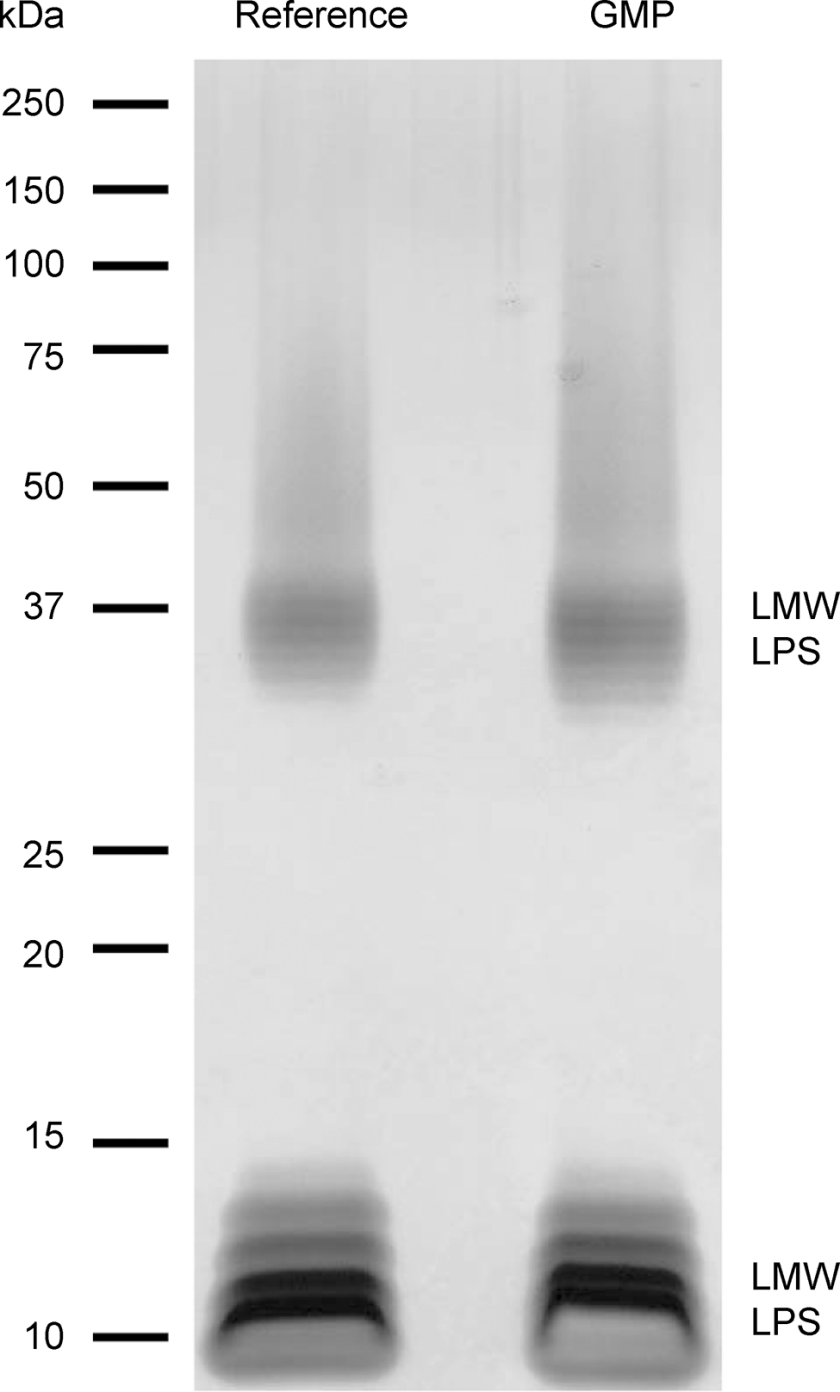
Silver stained SDS-PAGE gel of LPS extracted from 1790-GMMA reference and GMP batches.

**Fig 4:**
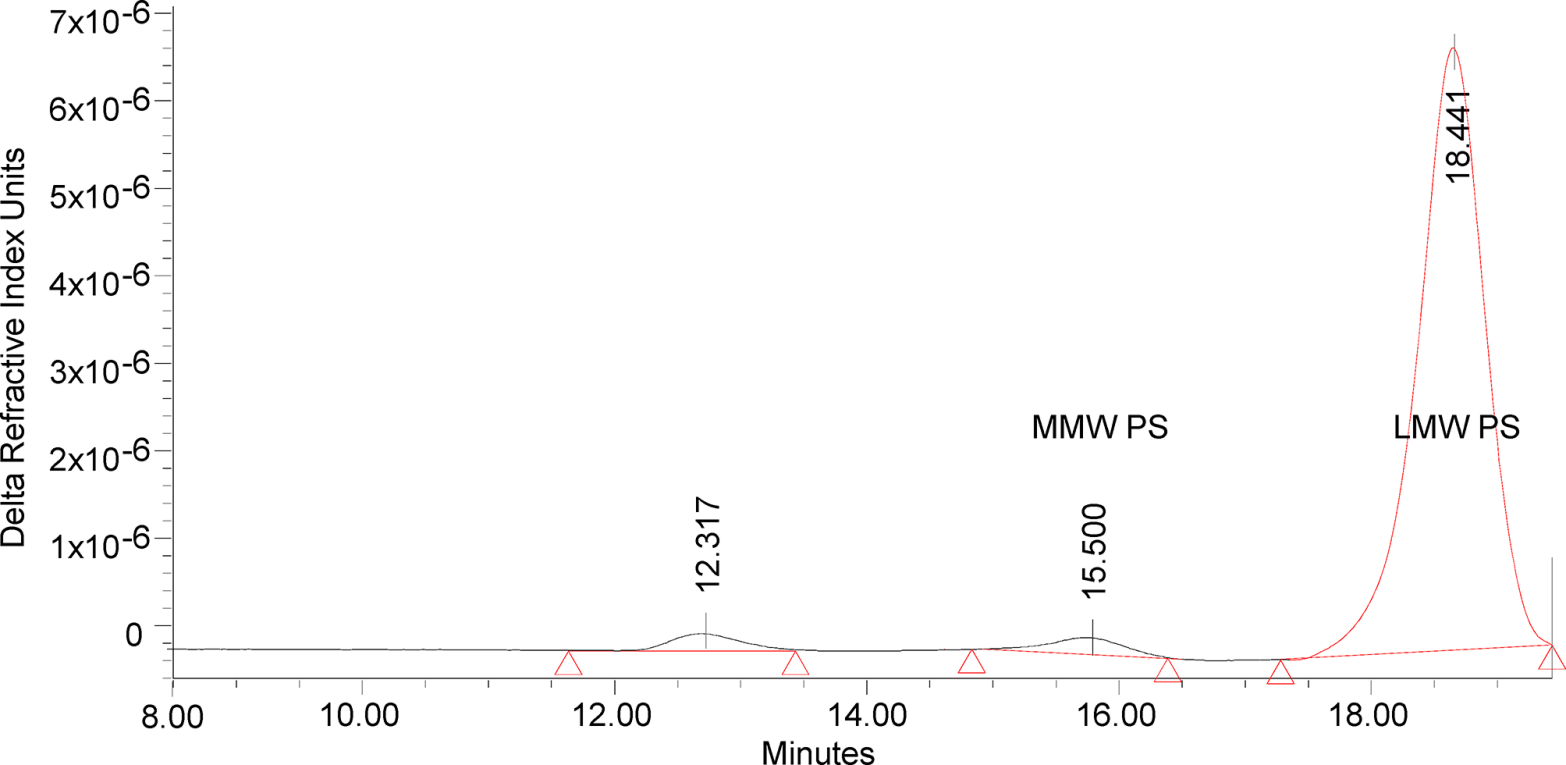
Separation of extracted O antigen from the 1790-GMMA reference batch by HPLC gel permeation chromatograph.

Silver stained SDS-PAGE of LPS extracted from reference and GMP batches showed an LPS ladder with a bimodal distribution (Fig 3). The predominant bands were low molecular weight LPS with up to 5 OAg repeats; medium molecular weight LPS was visible as a minor fraction. These data were consistent with analytical size exclusion chromatography of extracted OAg/core using refractive index detection (Fig 4). Again the dominant peak is low molecular weight polysaccharide and a less abundant medium molecular weight population. By this technique a small amount of high molecular weight polysaccharide was also detected. This size distribution of predominantly low molecular weight LPS in 1790-GMMA is markedly different to the size in the reference LPS derived from the parent strain without the LPS modification (1859-GMMA) that have an average of 33 repeats measured by NMR.

The composition of the LPS core was determined by HPAEC-PAD analysis. This method demonstrated that the molar ratio of galactose to glucose in the LPS present 1790-GMMA is 2:4, whereas in GMMA from the parent strain without the LPS modification (1859-GMMA) the ratio was 2:3 and similar to *S*. *sonnei* wild-type strains [38]. The change of glucose content in the LPS core of 1790-GMMA was confirmed by analysis of a third LPS core sugar, the terminal KDO. In 1790-GMMA and 1859-GMMA, the ratio of galactose to KDO was the same. The ratio of glucose to KDO differed confirming a higher glucose content in the LPS core of 1790-GMMA.

#### Lipid A structure

**Fig 5:**
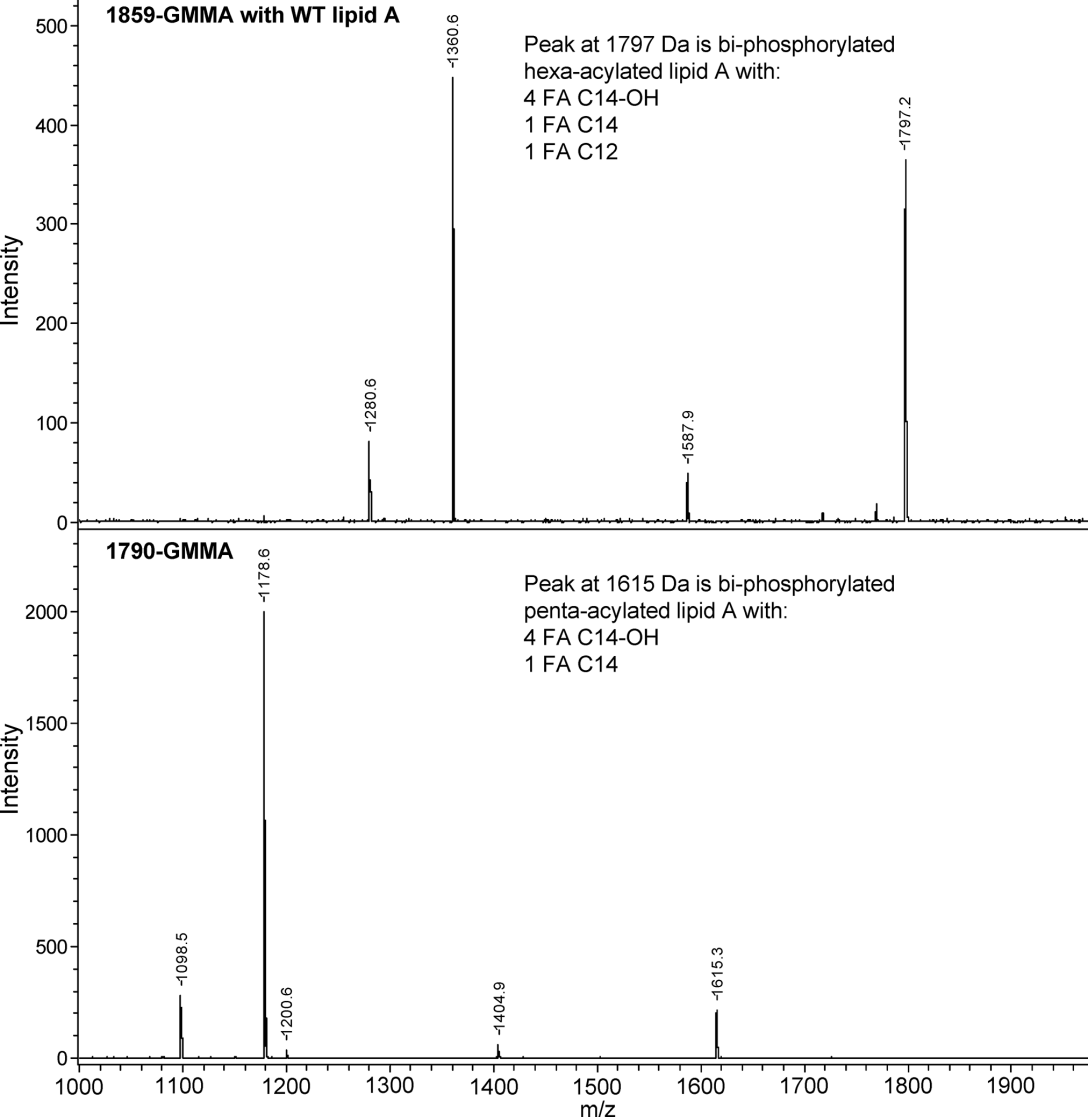
Mass spectra of lipid A extracted from 1859-GMMA (top panel) and 1790-GMMA (lower panel). The peak with the biggest m/z from the 1859-GMMA sample is the size expected for a hexa-acylated lipid A. No hexa-acyl lipid A was detected in the 1790-GMMA. The peak with the highest *m/z* in the 1790-GMMA lipid A sample has the mass expected for penta-acylated lipid A consistent with the Δ*htrB* mutation

The structure of the lipid A purified from the reference batch was determined by mass spectroscopy using MALDI-TOF (Fig 5). The recorded spectra showed penta-acylated lipid A corresponding to the highest peak and several other peaks due to its fragmentation (i.e. loss of one or more fatty acid chains), aggregate formation with sodium (+23 m/z) and de-phosphorylation (-80 m/z). No hexa- or hepta-acylated lipid A was detected by MALDI-TOF. For comparison, the MALDI-TOF profile of lipid A purified from the parental 1859-GMMA with *S*. *sonnei* unmodified (hexa-acylated) lipid A is shown.

#### IL-6 production in MAT

**Fig 6:**
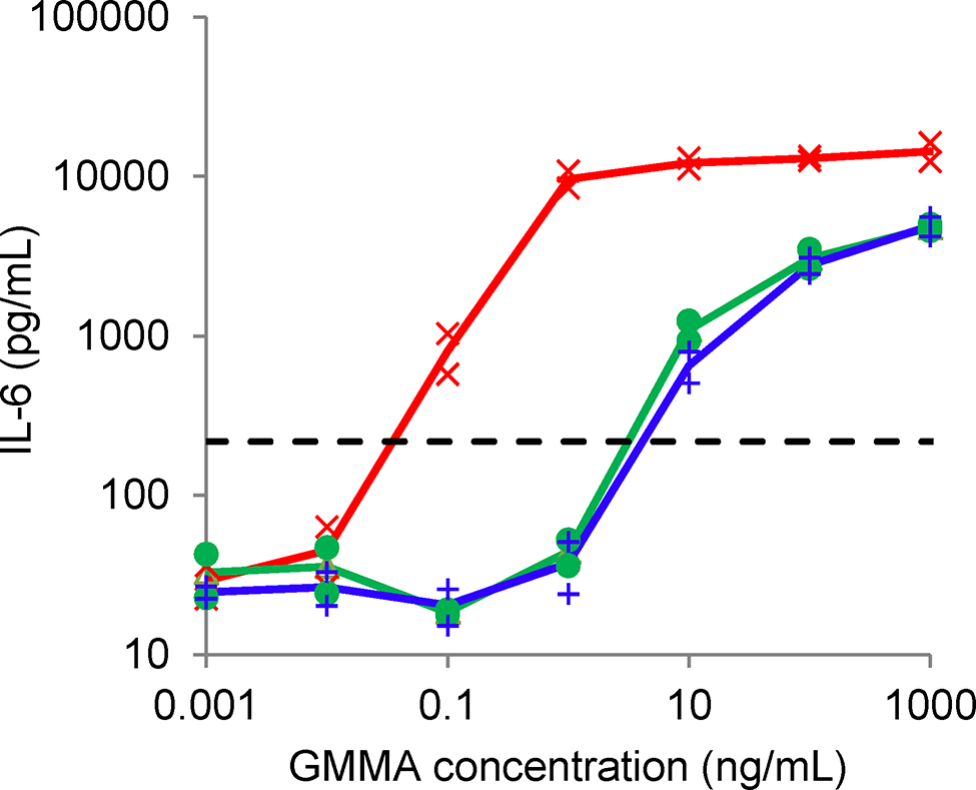
Monocyte activation test of 1859-GMMA (red X) and reference (green •) and GMP batches of 1790-GMMA (blue +) measured by the IL-6 production from human PBMC. Each sample was measured in duplicate. The horizontal dashed line is the IL-6 level ten times background. At this level, 1790-GMMA required 600x times the concentration of 1859-GMMA to give the same level of IL-6 release.

1859-GMMA containing unmodified LPS induced high levels of IL-6 release from PBMC, causing a 10-fold increase in IL-6 release over background at a concentration of 0.004 ng protein/mL, whereas 1790-GMMA, containing penta-acylated LPS, needed a 600x higher concentration (2.37 ng protein/mL) to cause the same IL-6 release (Fig 6).

### Intramuscular pyrogenicity test

**Fig 7:**
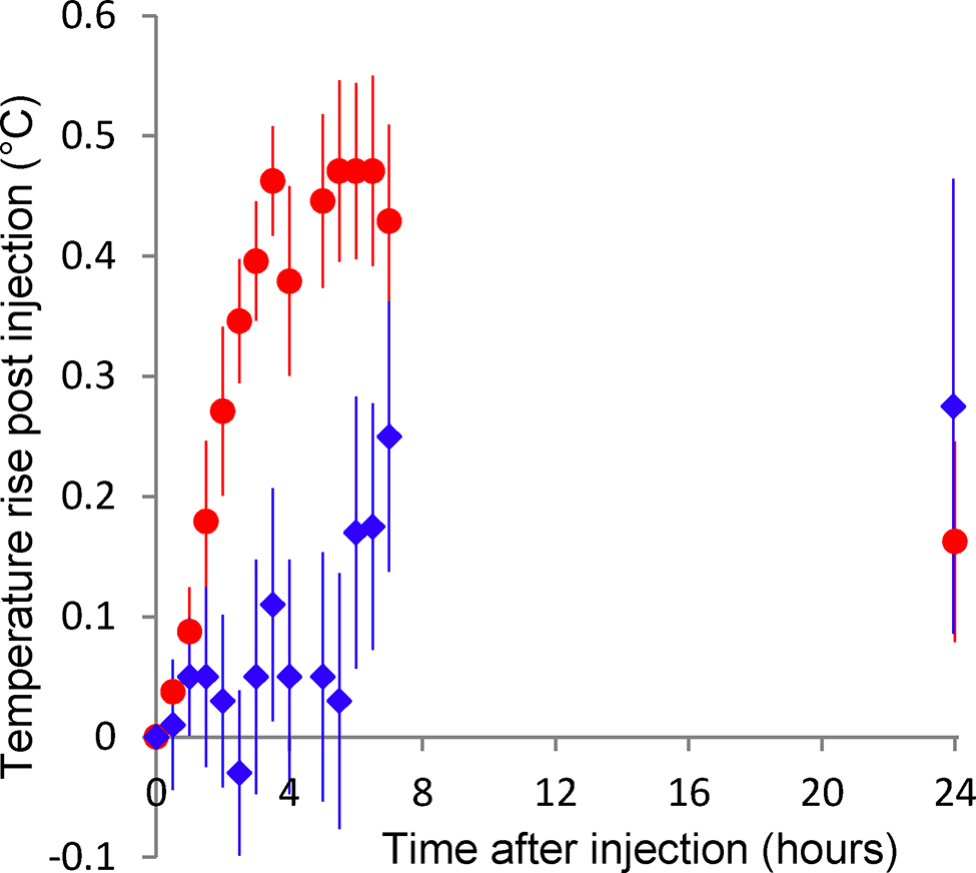
Mean temperature rise (mean of post vaccination–pre-vaccination temperature) in rabbits after an IM injection of 100 μg protein containing dose of 1790GAHB (red circles) or equivalent volume of physiological saline (blue diamonds). The vertical bars show the standard error of the mean. N = 12 for the 1790GAHB and 6 for the saline injected rabbits.

The average temperature rises observed at the different intervals up to 7 h, and at 24 h after vaccination in all 12 rabbits receiving GMMA vaccines (6 in the first study receiving the toxicology lot, 3 in the second study receiving toxicology lot and 3 receiving the clinical lot) are shown in Fig 7 compared to the average temperature rise of the 6 animals receiving saline. The average temperature rise in the vaccine group was still increasing at the normal 3 hour end point for an intravenous pyrogenicity test. After 5 h the average temperature rise in the control animals showed a significant increase and the difference between vaccine and control groups decreased. By 24 h the vaccine and control groups were not significantly different (vaccine group had a lower average temperature rise). Based on these results, 4 h was selected as the definitive time point.

Using the criteria developed as detailed in Materials and Methods, both the toxicology and clinical vaccine groups passed the pyrogenicity test without requiring a repeat assay. The mean "maximum temperature rise" over the first 4 h after administration of the toxicology lot and the clinical lot were 0.48 and 0.53°C, respectively. For the control group, the value was 0.27°C.

### Toxicology study

There was no mortality, no treatment-related clinical signs, and no changes in body weight or food consumption in rabbits treated with 1790GAHB vaccine or GAHB-Placebo. There were no treatment-related ophthalmological findings. No organ weight changes considered to be related to either vaccine or placebo administration were noted at day 44 or at day 56. (i.e. 2 or 14 days after the final vaccination). The vaccine was locally well tolerated by intranasal and intramuscular routes with no observed local reaction by IN route and very slight (erythema, edema) up to moderate local reactions (edema) observed in some of the rabbits after IM administration. The ID administration induced very slight to moderate local reactions (induration, erythema and edema) which were more pronounced in the 1790GAHB group than in the corresponding GAHB-Placebo group. Local reactogenicity had completely or partially resolved by the end of the two-week recovery phase at the IM injection sites but not at the ID site of injection. However, the tolerability to the vaccine by ID administration remained acceptable. Inflammatory changes of low severity and magnitude including changes in draining lymph nodes and spleen were noted upon histopathologic examination; these changes correlated with increases in C-reactive protein and fibrinogen and were consistent with this pharmacological response to an immunogen. The changes in clinical pathology parameters and the minimal to moderate microscopic changes had generally resolved by the end of recovery phase in the groups treated IN and ID while those seen in the group treated IM had slightly decreased indicating recovery from inflammatory changes was ongoing.

There was a statistically significant increase in temperature in the rabbits receiving IM 1790GAHB compared to the IM placebo groups. This was only seen for the IM groups and mainly in males. After the first IM vaccination there was an average temperature rise of 0.43°C for 1790GAHB vs 0.12°C for placebo (p = 0.009, t-test) at 2 hours and 0.64°C for 1790GAHB vs 0.38°C for placebo (p = 0.005, t-test) at 6 h compared to pre-vaccination. At 24 h post injection, there was no difference in these groups (0.21 and 0.22°C increases compared to the initial temperatures before vaccination, respectively). Similar temperature rises were seen in the IM groups following the 3^rd^ and 4^th^ injections (temperature increases of 0.44 vs 0.11 and 0.63 vs 0.33°C at 6 h compared to pre-vaccination for 1790GAHB vs placebo). A smaller but statistically non-significant increase was seen following the second immunization (0.28 vs 0.14°C at 6 h compared to pre-vaccination). While the differences were considered to be 1790GAHB-related, in view of the very low magnitude of the variation and the short period of increases, the effect was not considered to be toxicologically relevant.

### Irwin study

The Irwin study in rats to assess neurotoxic effects of IN vaccination following a single administration of 1790GAHB showed no relevant effects on a battery of behavioral and physiological parameters covering the main central and peripheral nervous system functions.

### Immunogenicity and potency in mice

**Fig 8:**
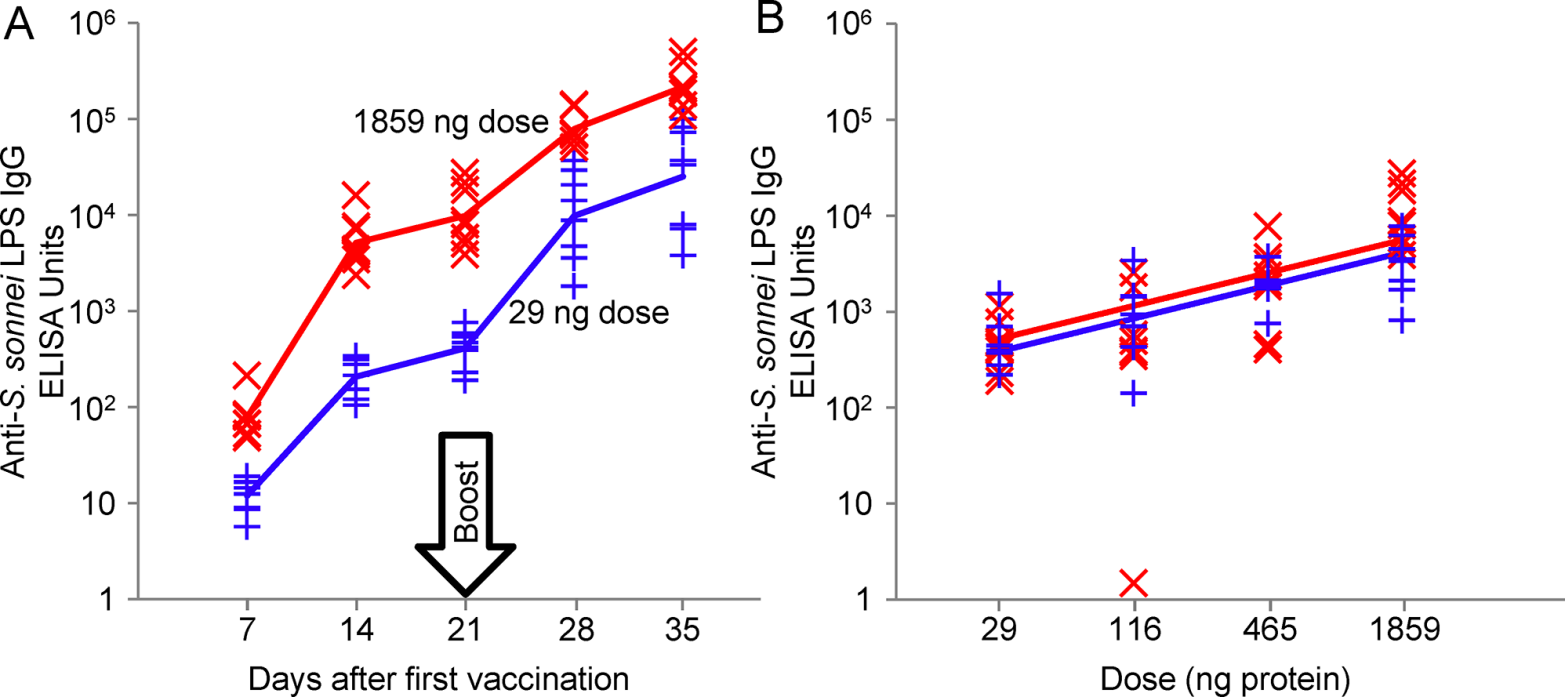
Antibody responses in mice. A: vaccinated at 3 week intervals with 1790GAHB containing 29 ng or 1859 ng protein. B: at day 21 following a single injection of 1790GAHB containing 29, 116, 465 or 1859 ng protein. This is the result from a potency study; half the mice were vaccinated with the appropriate doses of a formulation of 1790GAHB prepared from the reference batch of 1790-GMMA stored at -80°C (blue, +) and formulated 2 days before vaccination. The other mice were vaccinated with appropriate doses of the NVGH 1790GAHB vaccine stability batch (red, X). All doses contained the same amount of Alhydrogel in 0.5 mL buffered saline.

The initial immunogenicity study evaluated 7 different doses of 1790GAHB increasing 4-fold from 29 ng to 238 **μ**g protein (1.75 ng to 14.35 **μ**g of OAg) injected intraperitoneally and showed that the vaccine was highly immunogenic (Fig 8). Antibody was detectible at all doses after a single injection and was boosted following a second injection. 1.86 **μ**g of protein (0.11 **μ**g OAg) triggered the maximum antibody response. Based on these results, an immunogenicity protocol was developed to form the basis of potency tests and to assess stability over time as judged by potency. The final potency study design used four, 4-fold increasing doses of 1790GAHB from 29 ng to 1.86 **μ**g of protein in groups of 8 mice with serum IgG levels assessed by ELISA on LPS with the homologous OAg three weeks after a single immunization. A reference 1790GAHB preparation was freshly formulated for each potency study, and administered at the same doses as the test vaccine. The dose-response curves of the antibody levels elicited by test vaccine and reference standard were compared. Results of the initial potency study of the vaccine stability batch compared with the freshly formulated reference standard are shown in Fig 8. There was no significant difference in the slope or intercepts of the linear regression of the log transformed anti-LPS antibody on the log dose showing that the vaccine stability batch had the same potency as the freshly formulated reference material.

### Immunogenicity in Rabbits

**Fig 9:**
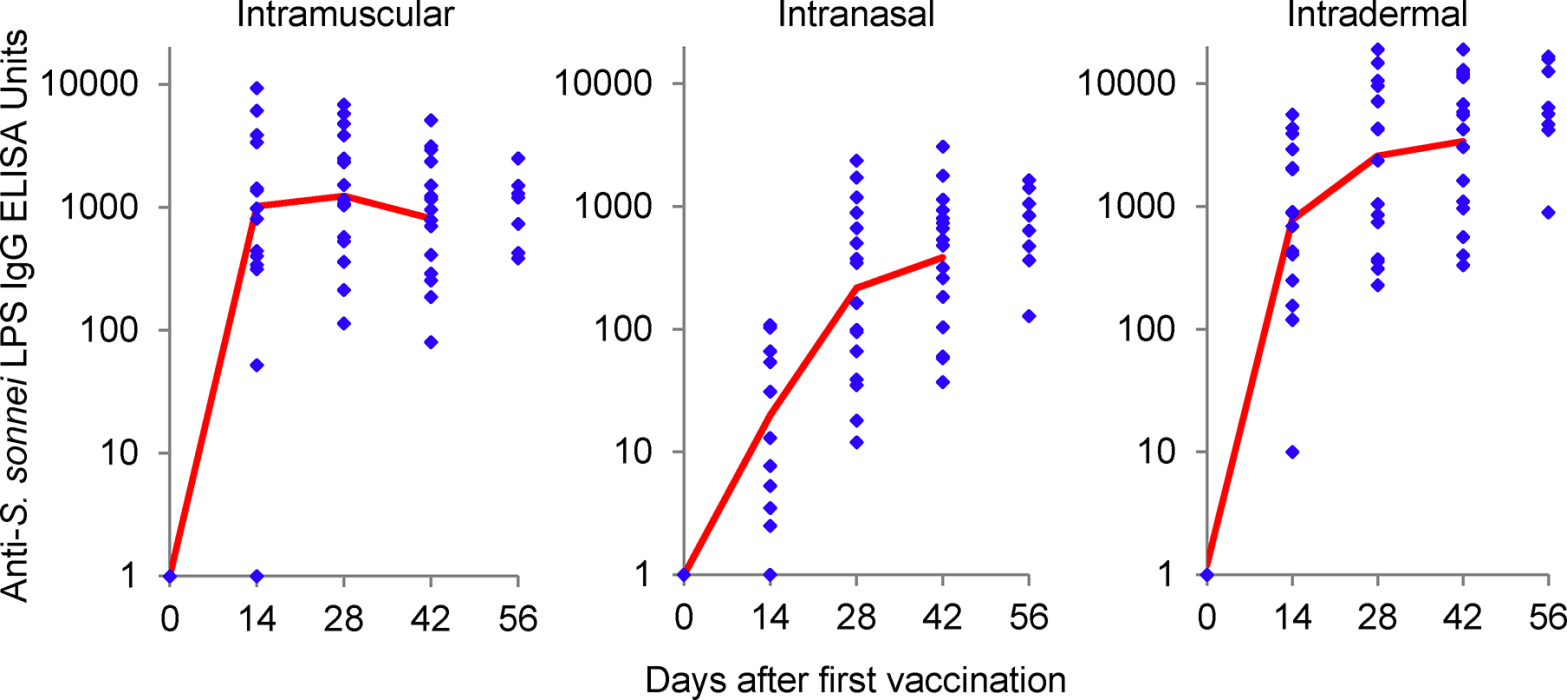
Antibody levels in rabbits vaccinated with 100 μg IM, 80 μg IN, or10 μg ID in the toxicology study. Antibody levels for each of the 12 rabbits per group are shown (dots) and group geometric mean for days 0 to 42 (line). Half of the rabbits were sacrificed at day 44 for assessment of immediate adverse effects. The antibody levels at day 56 reflect the levels in the remaining 6 animals in each group.

The IgG response in the rabbits from the toxicology study was assessed following each vaccination and at the final bleed. All three routes gave high levels of circulating anti-LPS IgG (Fig 9). The maximum response had been achieved 14 days following a single IM injection of 100 **μ**g dose of 1790GAHB. For the IN route, maximum antibody response took two immunizations. The circulating IgG anti-LPS levels 14 days after the final vaccination were not significantly different to the level achieved with IM delivery of the 100 **μ**g dose of 1790GAHB. The 10 **μ**g ID vaccination also gave an increase in response with subsequent vaccinations (Spearman rank test p <0.0001) but the effect was less pronounced than with the IN route. The final circulating anti-LPS IgG levels were significantly higher by the ID route than by the IM route (t test of log transformed antibody p = 0.002).

## Discussion

Although shigellosis is a significant health burden in travelers from industrialized countries, the major burden of disease is in residents of low and middle income countries. Thus, in addition to efficacy, low reactogenicity, and a good safety profile, vaccines designed for use in these populations should be manufactured for a low cost of goods and by a process that is readily scalable to very large numbers of doses. 1790GAHB has promise in each of these areas.

In an extension of laboratory scale production of *S*. *sonnei* GMMA [22], the scale up to 30 L pilot scale with a production strain of *S*. *sonnei* (NVGH1790) was straightforward, and using the same two step filtration process gave high purity GMMA in high yield and with good reproducibility between consistency and GMP runs. The production time from setting up the inoculum for fermentation to final purified GMMA was 3 days and thus, depending on the size of human dose, even a relatively small production facility (e.g. a 500 L fermenter) could produce in excess of 100,000,000 doses of vaccine per year.

While serum IgG levels correlated with protection seen following IM vaccination with an O antigen conjugate vaccine [21], in this paper immunogenicity in mice and rabbits is done not as an assessment of potential protection but as part of the required preclinical release and safety studies. 1790GAHB is highly immunogenic in mice and rabbits; low doses induced high levels of anti-LPS antibodies. Vaccination of mice with 29 ng protein/1.75 ng of OAg gave substantial serum antibody after a single injection with a geometric mean ELISA units of 207. Following a second injection 4 weeks later, antibody was boosted 121-fold. The high immunogenicity in mice following a single injection has facilitated the development of a relatively rapid potency test, currently being used to assess long term stability. This test supplements ongoing studies of the chemical stability of the O antigen. It will addresses the question of whether long term changes in GMMA structure, including components other than the modified LPS, may impact on the immunogenicity of the O antigen presented in the context of the formulated GMMA, regardless of any mechanisms of protection this vaccine may elicit in humans. This is a critical part of the regulatory requirements to specify a vaccine shelf life, even at this early stage of the clinical development plan.

At the doses tested in the rabbit toxicology study (100, 80, or 10 **μ**g of protein equivalent to 6.4, 5.1, or 0.64 **μ**g of OAg by the IM, IN, or ID routes, respectively) the antibody response peaked after the first dose IM, but showed increasing antibody on subsequent doses IN and ID. Interestingly the maximum antibody response measured in rabbits receiving the 10 **μ**g protein dose ID was significantly higher than in rabbits receiving 100 **μ**g protein IM. While the relevance of this response can only be judged following vaccination of humans, these results show that rabbits are a suitable species for a pre-clinical toxicology study. If there had been no or minimal response in the rabbits at the maximum dose chosen for human studies (10 **μ**g protein dose ID, 80 **μ**g protein dose IN, and 100 **μ**g protein dose IM) then this would have ruled out rabbits for the toxicology study.

Since GMMA are formed from the bacterial outer membrane and contain high concentrations of LPS and other stimulators of innate immunity, reduction of potential reactogenicity of GMMA and *in vitro* tests and animal models of reactogenicity were a priority for the preclinical development. Modification of the LPS to be penta-acylated was preferred due to the low stimulatory potential for human TLR4 [25,43]. As judged by mass spectroscopy of the extracted lipid A from 1790-GMMA, the introduction of the Δ*htrB* mutation resulted in penta-acylated lipid A with no trace of contaminating hexa-acylated lipid A (Fig 6), in accordance with our previous results with GMMA from OAg-deficient *S*. *sonnei* [25]. Consistent with this, the MAT using human PMBC stimulated with 1790-GMMA showed a markedly lower IL-6 production compared to PMBC stimulated with the parent GMMA with unmodified LPS. Importantly, the high concentrations of 1790-GMMA required to elicit IL-6 compared to wild type GMMA (used as a positive control) gave confidence that the 1790-GMMA are unlikely to be reactogenic in humans and that other components in GMMA (e.g. lipoproteins activating IL-6 production via TLR2 activation) are unlikely to be a significant sources of reactogenicity in the clinical production lots.

Three *in vivo* experiments show that the 1790GAHB formulation of the modified GMMA is likely to be acceptable for use as a vaccine: the rabbit toxicology study, the rabbit pyrogenicity study, and the Irwin study in rats. Rabbits are an appropriate species for the toxicology study since they gave a strong immunological response to the *Shigella* LPS by all three immunization routes and also they respond strongly to the LPS present in Gram-negative bacteria [44]. In this study, no local or systemic toxicologically relevant findings were made. There were some minor temperature increases in the group receiving the vaccine by IM route but these were transient and an average increase of only approximately 0.3°C above the placebo.

In the modified intramuscular pyrogenicity test using administration of a full human dose, a temperature rise of approximately 0.5°C was observed in the vaccine groups of the toxicology and clinical lots until 4 hours following administration. In view of the low average temperature variation and of the short duration of the increase (< 24 h), the method demonstrated the appropriate sensitivity and ability to measure potential pyrogenicity of products from Gram-negative bacteria that inherently contain LPS as structural component. Thus, the modified Ph.Eur. pyrogenicity test method is regarded a promising alternative for this type of products. While the pyrogenicity test described here will further be used for release testing of GMMA during development, the investigation of correlation to clinical data coming from human vaccine trials and the ability of the test to reliably detect substandard lots will be the next steps.

As for the rabbit toxicology studies, the Irwin test in rats showed no evidence that IN immunization with 1790GAHB will cause neurological problems.

Thus, the production, lot release, immunogenicity and pre-clinical safety tests were satisfactory and *S*. *sonnei* 1790GAHB vaccine is in dose-escalating clinical trials via IM, ID, and IN routes. Critically, only these clinical studies can validate *in vitro* and animal studies as a predictor of the safety profile, especially with regard to reactogenicity. The current trials form the start of a clinical development plan to address the immunogenicity of the O antigen in humans and ultimately, if the vaccine is not only immunogenic but induces protection, the relatively importance of different classes of antibody (e.g. sIgA vs IgG) and different modes of immunity (e.g. mucosal vs systemic) to protection from shigellosis induced by this vaccine.
